# Causal Inference With Observational Data and Unobserved Confounding Variables

**DOI:** 10.1111/ele.70023

**Published:** 2025-01-21

**Authors:** Jarrett E. K. Byrnes, Laura E. Dee

**Affiliations:** ^1^ Department of Biology University of Massachusetts Boston Boston Massachusetts USA; ^2^ Department of Ecology and Evolutionary Biology University of Colorado Boulder Boulder Colorado USA

**Keywords:** causal inference, causality, correlated random effects, endogeneity, mixed models, observational data, omitted variable bias, panel regression, structural causal model

## Abstract

Experiments have long been the gold standard for causal inference in Ecology. As Ecology tackles progressively larger problems, however, we are moving beyond the scales at which randomised controlled experiments are feasible. To answer causal questions at scale, we need to also use observational data —something Ecologists tend to view with great scepticism. The major challenge using observational data for causal inference is confounding variables: variables affecting both a causal variable and response of interest. Unmeasured confounders—known or unknown—lead to statistical bias, creating spurious correlations and masking true causal relationships. To combat this omitted variable bias, other disciplines have developed rigorous approaches for causal inference from observational data that flexibly control for broad suites of confounding variables. We show how ecologists can harness some of these methods—causal diagrams to identify confounders coupled with nested sampling and statistical designs—to reduce risks of omitted variable bias. Using an example of estimating warming effects on snails, we show how current methods in Ecology (e.g., mixed models) produce incorrect inferences due to omitted variable bias and how alternative methods can eliminate it, improving causal inferences with weaker assumptions. Our goal is to expand tools for causal inference using observational and imperfect experimental data in Ecology.

## Graphical abstract

As ecology tackles progressively larger problems, we are moving beyond the scales at which randomised controlled experiments are feasible. Using observational data for causal inference raises the problem of confounding variables, those affecting both a causal variable and response of interest, which can create bias when not measured and omitted from statistical models. Here, we show how ecologists can harness methods from other disciplines that handle this omitted variable bias to produce better causal inference from observational data at scale.

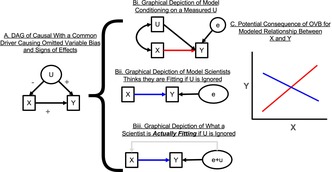

## 1 Introduction

As Ecology advances to address problems at scales from the continental to global, we are putting our theories to the test like never before with unprecedented streams of data. With these observational data streams, we desire to answer questions about causal relationships to either test theory at scale or inform ecosystem management. Classically in Ecology, understanding causal relationships has been the domain of experiments. Experiments, however, have limitations for generalising to large scales or contexts beyond study conditions. Scaling up inference will therefore require us to responsibly seize the opportunity of large‐scale observational data. Our ability to test hypotheses about causal relationships in observational data is limited, however, by two fundamental challenges: the complexity of nature and the limits of our own imaginations.

**FIGURE 1:**
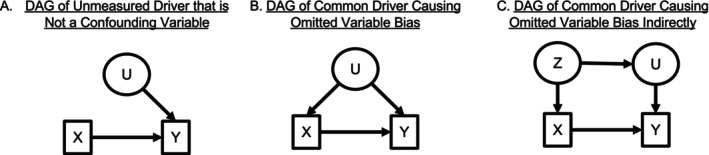
Directed Acyclic Graphs showing scenarios where unobserved variables either do not influence model results or could create problems due to confounding. A response variable of interest (*Y*) is caused by both a measured variable (*X*) and an unmeasured variable (*U*). In (A), *X* and *U* are uncorrelated, and thus the lack of inclusion of *U* in a statistical model would increase the standard error of the estimate (decreases model precision) but would not lead to bias in the effect of *X* on *Y*. However, if *U* also drives *X* as in (B) or if *U* and *X* are driven by a common driver *Z* as in (C), then omitting *U* from a statistical model causes omitted variable bias in the estimate of the effect of *X* on *Y*. Both (B) and (C) are examples of systems where the confounding common causes (*U* and *Z* respectively) must be controlled for in order to make unbiased causal inferences.

**FIGURE 2:**
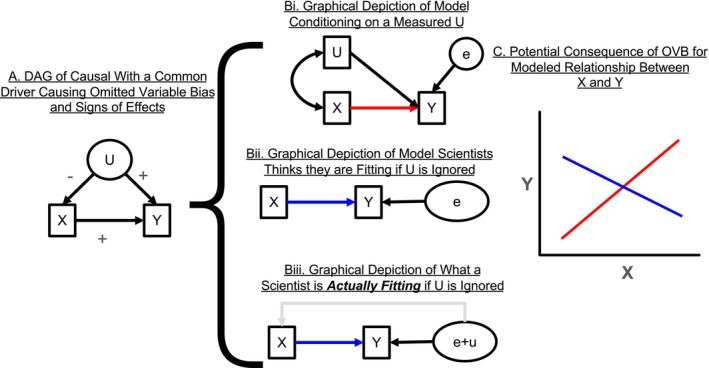
A visualisation of Omitted Variable Bias and the consequences for causal inference. (A) shows a DAG of a system where *X* has a positive effect on *Y*, and a confounding variable *U* has a positive effect on *Y* but a negative effect on *X*. Throughout, *unobserved* (i.e., unmeasured) variables are shown in in ellipses, such as the variable *U* and the error term *e* in panel B. (B) illustrates different *estimations* of the DAG in (A) using a path analysis. See Box 1 for a brief explanation of key differences between DAGs and path diagrams. Again, we assume *U* is unmeasured. In (Bi), we assume we can measure and control for *U*, as represented by the double‐headed arrow between *U* and *X*, which represents the correlation between the two accounted for by the model. The unmeasured variable *e* is the residual sources of variation which as assumed to correlate with neither predictor. The red arrow represents the estimated path. In contrast, (Bii) and (Biii) are the reality—where we do not have a measurement of *U* and do not control for it in the path model. The researcher thinks they are fitting the model in (Bii) but instead they are fitting the model in (Biii), where the error term is not e alone, but rather the sum of e and variation due to the omitted variable *U*. Because of this, there is a directed path from the error term of the model to *X* (and thus *X* is endogenous). (C) shows the estimated relationships resulting from the models in (Bi) versus (Bii). The lines represent the estimated relationship between *X* and *Y* from their respective models. The red line is the true causal relationship, as estimated from (Bi) and the blue line contains omitted variable bias from not accounting for the confounding variable U as estimated by the model in Bii/Biii.

First, nature is complex! Consequently, numerous **confounding variables**—variables affecting *both* a cause and outcome of interest (Figure 1B,C), as opposed to variables only influencing only the outcome (Figure 1A), exist in every system. Confounding variables can lead to incorrect estimates of causal effects when not measured and controlled for in statistical analyses. Failing to control for confounding variables leads to **bias** in our statistical estimators; the estimates they yield will not be equal to their true value (Figure 2). A simple solution for bias from confounding variables is to statistically control for these confounders. Yet, this control requires knowing and measuring all confounding variables. Collecting the data to measure and account for each and every one is likely impossible, assuming we even know all of confounders. For example, when studying plant competition, measuring all the relevant soil properties is challenging due to financial and time constraints. Missing data on confounders is also common with long‐term survey data. Consider using historical measures of fish abundance to study the impacts of changes in biogenic habitat availability without measurements of fishing pressure during the same time‐period. If fishing pressure lessened while habitat decreased, we might conclude that habitat has a negative effect on fish.

Second, as humans, we are limited in our ability to imagine how the different elements of complex ecological systems are causally related. Thinking through the entire natural history of a system to design an analysis that accounts for all confounding variables is really hard, even for the most experienced researchers. We also might not know which confounding variables are most important to measure and control for to eliminate bias. As a result, causal inference from observational data is often dismissed as impossible, prompting the saying “correlation is not causation.” Thus, dealing with the problems created by not controlling for unmeasured confounders in our statistical analyses is a first‐order challenge for inferring causation from observational data (Figures 1 and 2).

Omitting known but unmeasured, or unknown and unmeasured, confounding variables from a statistical analysis creates Omitted Variable Bias or **OVB** (Rinella, Strong, and Vermeire 2020; Wooldridge 2015). OVB results in estimators yielding the incorrect magnitude—or even sign—of estimates (i.e., biased estimators). OVB can create spurious correlations or mask true causal relationships. OVB differs from measurement error in predictor variables, which produces a consistent bias towards zero and can be corrected for or modelled (McElreath 2020 chapter on measurement error; Schennach 2016). With OVB, we cannot know the magnitude or direction of bias without knowing all confounding variables in a system. As measuring, controlling for, and even knowing all potential confounding variables is nearly impossible in complex ecological systems (reviewed in Dee et al. 2023), we are always going to miss something, threatening the validity of causal inferences.

*Do challenges from OVB mean that we should avoid using observational data for causal inference?* No! Rather than discounting and abandoning observational data for causal inference, we suggest that ecologists consider adopting well‐established techniques from other disciplines, including economics, sociology, epidemiology, and computer science, that offer solutions (Angrist and Pischke 2008; Heckman 2000; Hernan and Robins 2023; Holland 1986; Imbens and Rubin 2015; Morgan and Winship 2015; Pearl 2009; Robins 1989; Rubin 1974, 2005). These techniques aim to replicate, or get as close as possible to, a randomized experiment. Because these fields cannot always do experiments for logistical or ethical reasons—for instance, it is not ethical to force a person smoke cigarettes daily to quantify the causal effect of smoking on dementia (Hernan and Robins 2023)—they have been developing tools to handle OVB for decades. Yet, these tools have been largely absent from the ecologist's toolbox until relatively recently, with some exceptions (Arif and MacNeil 2022b, 2023; Butsic et al. 2017; Dee et al. 2023; Dudney et al. 2021; Grace and Irvine 2020; Larsen 2013; Larsen, Meng, and Kendall 2019; MacDonald and Mordecai 2019; Rinella, Strong, and Vermeire 2020; Simler‐Williamson and Germino 2022). If we, as a discipline, are to move to more widespread use of observational data for causal inference, we need to carefully consider the problems of OVB, the techniques we can use to mitigate them, and their assumptions.

Here, we aim to provide a guide to readily accessible methods to cope with omitted variable bias (OVB) for ecologists. We begin describing the status quo for how ecologists most often deal with OVB. We then review tools for identifying potential sources of OVB before conducting a study or analysis, building on the foundation of using directed acyclic graphs (Arif and MacNeil 2023). To illustrate the techniques, we present a motivating example of studying the effect of temperature on marine snail abundances. With this example, we outline sampling and statistical designs for dealing with OVB and demonstrate them with simulations. We compare the conclusions that would be drawn from the typical approaches an ecologist might take (e.g., mixed effect models, Bolker et al. 2009) to other statistical designs that can more adequately control for omitted variables. While common approaches produce statistically biased results, our simulations demonstrate the utility of the statistical designs that are underutilised, if not novel, in Ecology. We provide guidance for choosing among these designs along with a hands‐on tutorial with R code for prospective users (see Supporting Information S7 for worked examples). This paper complements recent reviews in Ecology of quasi‐experimental methods (Arif and MacNeil 2022b; Butsic et al. 2017; the appendices of Dee et al. 2023) by expanding on cross‐sectional and panel regression designs (see study design section for definitions) accounting for OVB.

## 2 How Are Ecologists Coping With Omitted Variables Bias?

Confounding variables and omitted variable bias are commonly dealt with in one of five ways in Ecology. First, Ecologists use randomised controlled experiments. In an ideal randomised controlled experiment, the effect of confounding variables is eliminated when design assumptions are met. We can interpret observed effects of manipulations as causal [but see Kimmel et al. (2021) on why this can be difficult in practice, particularly in the field]. Random assignment of treatments (e.g., nitrogen addition) to units (e.g., plots) means that the treatment and control groups have the same level of any confounders on average. However, randomised controlled experiments are not always feasible, especially at large scales, and can impose experimental conditions that create artefacts which make generalising to natural systems difficult (Ruesink 2000; Stachowicz et al. 2008; Wolkovich et al. 2012). Second, in observational studies, ecologists attempt to remove effects of confounding variables by measuring and controlling for them in statistical analyses such as multiple regression. Yet, as described above, measuring all confounders is often impossible—particularly in retrospective analyses where existing data have been collected for another purpose. Moreover, all potential confounders might not be known. Third, ecologists use mixed‐effect models. These mixed models fold unmeasured, cluster‐level variables into random effects (Bolker et al. 2009; Harrison et al. 2018; Schielzeth and Nakagawa 2012). As discussed below (see section on statistical designs), if random effects are correlated with causal drivers of interest, random effect estimators are biased. Fourth, Ecologists sometimes make causal claims rooted in their knowledge of natural history. These claims can be problematic due to a lack of transparency and potential for incorrect statements about effect sizes; even the most accomplished naturalist can have gaps in their understanding of a system. Fifth, ecologists often qualify their results verbally to avoid making causal claims—even when their research focus is causal understanding, rather than description (but see Laubach et al. 2021). This practice muddies the waters and can create confusion over whether an author is claiming an association or implying causation while allowing themselves plausible deniability. We feel that, given our current need to understand causal relationships at large scales, these solutions are inadequate. In the worst case, they can lead to misleading inferences. So, we turn to solutions from other disciplines to the problem of omitted variable bias to augment our toolkits.

## 3 Using Causal Diagrams to Clarify Causal Assumptions and Ferret Out Omitted Variables Bias

Causal diagrams (a.k.a. Structural Causal Models from Pearl 1995; see Grace and Irvine 2020; Arif and MacNeil 2023 for introductions for Ecologists) are the first tool for identifying omitted variable bias (Arif and MacNeil 2023; Pearl 1995; Pearl, Glymour, and Jewell 2016). Causal diagrams in the form of directed acyclic graphs (DAGs, see Box 1 and Supporting information S1) visualise our understanding of causal relationships and confounding variables within a system. In doing so, DAGs transparently clarify assumptions behind our causal claims about relationships inferred from data and show potential sources of bias from confounding variables and other types of variables (see Box 1). Critically, DAGs are assumed to include all common causes of a cause and response of interest, including all measured and *unmeasured* confounding variables (Pearl 1995). We suggest drawing DAGs before conducting a causal analysis—and, if possible, *before* data collection to inform which covariates might be confounding to measure.

BOX 1 **An overview of directed acyclic graphs for causal analysis and detecting confounders.**

Causal diagrams (e.g., Figure 1), called Directed Acyclic Graphs (DAGs), help to determine where confounding variables might cause omitted variables bias and, in turn, to identify solutions in terms of sampling and statistical designs. In DAGs, assumed causal relationships among variables are implied by arrows with the direction of the arrows representing the direct of the causal effect. If the value of a causal variable of interest changes (e.g., via manipulation, exposure, or a natural process), there will be a concomitant change in the response variables it affects. In DAGs, these relationships are non‐parametric, without functional form. Critically, for a DAG to be complete, it should include both measured and unmeasured confounding variables. We represent observed variables that can be or have been measured with boxes (e.g., *X* and *Y* in Figure 1), and unobserved (i.e., unmeasured) variables within ellipses (*U* and *Z*).

DAGs help identify how and when to control for confounding variables. With a DAG, confounding variables can either be visually obvious or identified via software analysing conditional independence among variables (e.g., Textor et al. 2016). Confounding variables can be included and controlled for directly in a statistical model or by controlling for a “child node” of that confounding variable (e.g., *U* as a child of *Z* in Figure 1C or see Figure S1 for examples). Controlling for confounding variables helps satisfy the **back‐door criterion** (Pearl 1995, Figure S1A) for causal identification. That is, including variables that block all paths flowing from a *common cause* (e.g., *U* in 1B or Z in 1C) to both a causal variable of interest (*X*) and its response (*Y*) to “shut the back door” for causal information to flow between X and Y having nothing to do with their causal relationship. By “shutting the back door”, the estimate of the relationship between *X* and *Y* is then causally identified. Without controlling for confounding variables or others that block their influence in an analysis (e.g., see Figure S1), omitted confounders will cause OVB. A DAG also reveals where it is not possible to “shut the back door” due to unmeasured confounders, showing that other approaches—those presented in this manuscript, instrumental variables, Pearl's “front door criterion,” etc.—are needed (Pearl 2009; Bellemare, Bloem, and Wexler 2024).

DAGs also show what variables should *not* be included in an analysis, such as those causing collider bias or are otherwise bad controls (e.g., conditioning on mediator variables on the causal path from *X* to *Y*). Collider bias occurs when evaluating a relationship between two variables, but conditioning on a variable they both cause. For example, conditioning on plant abundance when analysing the relationship between disturbance intensity and herbivory intensity, but both causes plant abundance. In contrast, model selection metrics such as AIC—for predict versus causal aims—might favour including colliders or other bad controls (Arif and MacNeil 2022a). Here, we focus on bias from omitting important variables rather than including the wrong variables. This topic has been amply covered elsewhere (see McElreath 2020, Chapter 6; Laubach et al. 2021; Griffith et al. 2020).

DAGs are different than path models or other graphical depictions of statistical models more common in Ecology (e.g., Structural Equation Models). Both seek to show directional connections between variables but differ in several ways. (1) DAGs only represent causal relationships; path models can be causal or not. Path models can be used for non‐causal aims and include unexplained correlations as double‐headed arrows and other elements of error generating processes. (2) DAGs must include all common causes of the causal variable of interest and response for causal identification. (3) DAGs are non‐parametric and not tied to an estimation approach; path models represent an algebraic representation of a system in the form of a statistical model. (4) Path models can include feedbacks and cycles, whereas DAGs are acyclic (see Suppoting Information S2 for a discussion of feedbacks and DAGs). To show the links between DAGs and statistical models, here we present both DAGs and path models (e.g., Figure 2).

As applied researchers, we have found that DAGs, paired with robust statistical approaches for causal inference, clarify our own thinking and communication about ecological systems. Further, when multiple different theories suggest different DAGs, they can still be used to identify potential sources of confounding for analyses. For researchers interested in exploring possible DAGs or evaluating DAGs given some constraints, we refer readers to the field of causal discovery (Glymour, Zhang, and Spirtes 2019).

After building a DAG (see Box 1), one can determine potential sources of omitted variable bias, including from unmeasured confounding variables (e.g., *U* in Figure 1B), or potential “back doors” for confounding variation to flow between the causal variable and response variable (Pearl 2009). Said another way, omitting a confounding variable like *U* in Figures 1B and 2 that influences both *X* and *Y* in a statistical analysis means that it is folded into a statistical model's error term, along with random sources of error. Figure 2 illustrates the consequences of failing to control for *U*: the model's error term and causal variable are correlated, thereby producing an incorrect estimate of the effect of *X* on *Y* (Figure 2). This correlation is due to the causal variable (*X*) being **endogenous**—it is affected by elements in the error term. This endogeneity problem violates the assumptions of the Gauss‐Markov theorem and its extensions (Wooldridge 2015) and underlies OVB (Abdallah, Goergen, and O'Sullivan 2015; Antonakis et al. 2010).

To provide an example, consider studying the effect of nitrogen availability (*X*) on plant biomass (*Y*) across multiple fields, but nitrogen availability (*X*, as in Figure 2A) depends on field soil characteristics (*U*), and field soil characteristics also drive plant biomass (*Y*). If field soil characteristics were omitted from a statistical analysis, then (1) the effects of soil characteristics would be included in the error term of that model (Figure 2Biii) so that (2) nitrogen is no longer **exogenous** (external to the system of interactions) but instead endogenous, and (3) the effects of field‐level soil characteristics are misattributed to nitrogen, leading to incorrect estimates of nitrogen on plant biomass. Therefore, the estimate of the nitrogen effect will be wrong: different from the true effect in magnitude or even sign (Figure 2C). As discussed below, including field as a random effect does not resolve this problem. If we had drawn a DAG, we could have seen where endogeneity problems like this occurred and identified options to address them.

Finally, DAGs also justify choices of control variables; they make *transparent* the assumptions a researcher makes about how a system works for the readers of their work. However, DAGs, like our understanding of a system, can be incorrect or not include unknown confounding variables. While they provide a useful tool, a DAG only represents a researcher's *current understanding* *and own assumptions* about the causal relationships within a system. Even without the correct DAG, recognising the possibilities for unmeasured confounders enables leveraging complementary approaches that lessen our reliance on a perfectly correct DAG to control for confounders. Using a case study of snails influenced by temperature, we review these approaches that combine observational sampling designs with statistical designs to control for *unobserved* and potentially *unknown* confounding variables.

## 4 Case Study: A Problem of Omitted Snails

**FIGURE 3:**
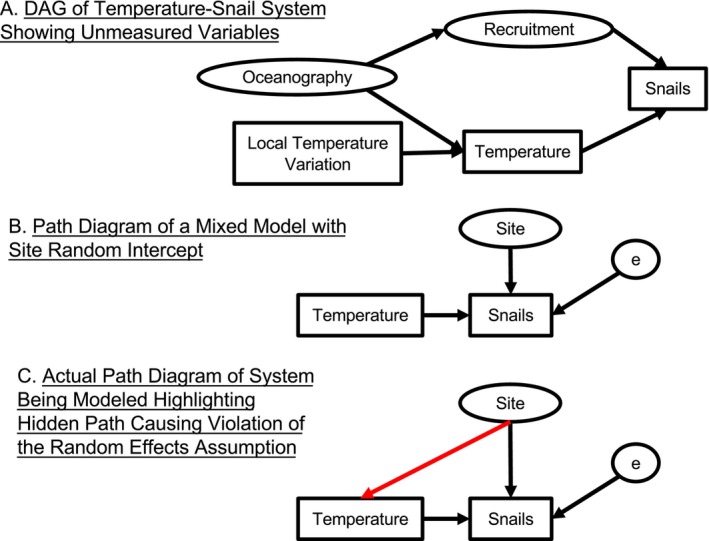
A causal diagram describing the controls of snail abundance in an intertidal ecosystem and path models of a random effects models ignoring the random effects assumption. In the system in (A), oceanography drives both temperature and recruitment, and both drive snail abundance. Temperature, however, is also driven by local influences as well. This could be variability in plot‐level temperature within a site—i.e., sources of variation in microclimate—or site‐level temperature variability over space or time uncorrelated with local oceanography, recruitment, or other site‐ or plot‐level confounders. The mixed effects model with a random effect for site in (B) assumes that there are no site‐level drivers of temperature and does not account for the relationship between site and temperature. Site effects here are site‐level residuals drawn from a normal distribution as in Equation (3). As error variables are unobserved, we include them in an ellipse as with other unobserved variables. Thus, the effect of temperature on snails is confounded by any correlated site‐level drivers that correlate with temperature at the site level. The assumption of no relationship between site and temperature contrasts with the violation of endogeneity highlighted in (C) showing that temperature is indeed at least partially driven by site. When we fit a mixed model, the red path in (C) is not included, creating an endogeneity problem and violating the Random Effects assumption. Said another way, our site‐level random effect is not endogenous, and thus the random effects estimator is biased (see simulations below and Figure 6 to see the biased estimates produced by a random‐effects model).

To illustrate the empirical challenges of unobserved confounding variables and potential solutions, we consider a marine benthic ecosystem modelled after the Gulf of Maine, USA, where a researcher aims to study the causal effect of temperature on snail abundance. They hypothesize that temperature affects snail metabolic and mortality rates and wish to estimate the effect of temperature on snail population abundance. Snail abundance is also driven by recruitment, in part influenced by regional oceanography (i.e., the flow of major currents which across space). Oceanography drives both water temperature and recruitment patterns (Broitman et al. 2005; Yund et al. 2015). We assume that snail abundance and temperature were measured at several sites but not recruitment or oceanography. Thus, recruitment and oceanography are unobserved confounding variables. Estimates produced from a statistical analysis of just the temperature‐snail relationship will almost certainly be wrong. Even if the researcher had measured recruitment and added it to a regression, other confounding variables could lurk and omitted variable bias remains a real possibility. The estimated effect of temperature on snails could still be incorrect. Fortunately, our researcher drew a DAG (Figure 3A) and recognised that temperature at the scale of a single observation was also influenced by local variation (e.g., from many sources of microclimatic variability). They realised that they could control for both observed and unobserved confounding variables with appropriate sampling and statistical designs.

## 5 Sampling Designs That Enable Statistical Methods to Cope With Omitted Variable Bias

**FIGURE 4:**
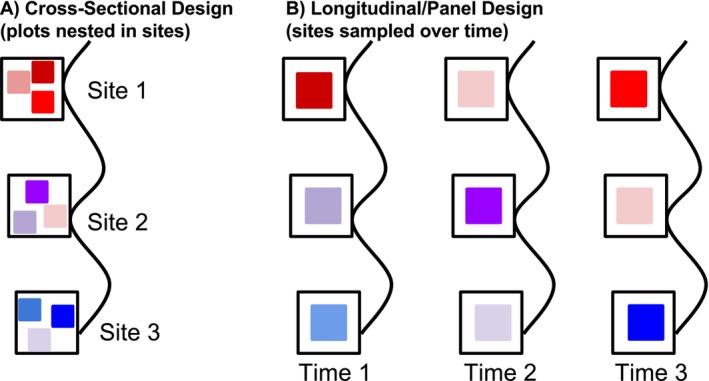
Visual examples of hierarchical study designs with plots nested within sites sampled at one point in time in A and through time in B. This figure shows sites distributed along a coastline with a corresponding thermal gradient, with one or more plots sampled within each site, depending on the design. Open squares are sites. Closed squares are plots within sites. Colour of square is proportional to temperature, with red and blue signifying warm and cold respectively and other colours placed on a gradient between them. These sampling designs therefore have variation across space, as in the cross‐sectional sampling design in A, or in both space and time as in B. which shows longitudinal or panel data, where the same plots within sites are observed through time. The sampling design in (A) can allow researchers to study temperature variation within sites as well as between sites. The design in (B) enables a researcher to leverage variation in space and time, including examining variation within sites through time.

Multiple sampling designs for data collection enable the use of statistical designs that can address omitted variable bias from confounding variables that vary across space, time, or both. A key feature in these sampling designs is some **hierarchical** or **clustered** structure to the data. Clustered data is often also referred to as a hierarchical or nested sampling design (Gelman and Hill 2006). We use these terms interchangeably. The nesting of multiple observations within a cluster or group (e.g., site) can allow the causal variable of interest to vary across replicates within a cluster while the confounder varies between clusters at the cluster level (Figure 4). This within‐between partitioning of variation enables us to control for confounding variation.

Clustered sampling designs can take several forms and generate different types of variation to study. First, a sampling design could include multiple plots sampled within sites at a single point in time (Figure 4A)—called a **cross‐sectional design**. When sites span environmental gradients with variation in a causal variable (e.g., temperature differences), confounding variables also vary across these spatial gradients. For instance, a spatial gradient in temperature across sites also reflects the spatial gradient in oceanography, confounding the effect of temperature and snails. However, with data collected from a cross‐sectional sampling design of plots *within multiple* sites, we can use variation in plot‐level temperature *within* sites to remove the confounding effects of between site differences (e.g., site‐level oceanographic features).

Second, one could sample the same plots or sites repeatedly through time (Figure 4B) creating **longitudinal** or **panel data**. Panel data generates variation within sites through time; it enables the use of approaches that removes confounding variation between sites, enabling causal inference on within‐site (or plot) variation through time. Developing an understanding of how cross‐sectional and panel data structures, and extensions (Box 2 and Supporting Information S3), can be used with statistical designs to remove confounding variation is key to confronting OVB.

## 6 Statistical Designs to Cope With Omitted Confounders

With hierarchical or clustered (hereafter clustered) data and a DAG in hand, we turn to well‐established statistical designs to control for unobserved confounding variables. We emphasise the term ‘*designs*’ over ‘*methods*’ because one could implement these statistical designs using a variety of estimation approaches (e.g., linear regression, generalised linear models, as a part of structural equation models, or with Bayesian techniques). These designs (see Table S1 for all design equations and definitions of terms) have different costs and benefits and differ in their assumptions required for interpreting an estimate as a causal effect (see Table S2). Yet, most of the following designs—with the exception of random effects models as shown below—allow us to flexibly control for both known and unknown confounding variables (see Angrist and Pischke 2008; Dudney et al. 2021; Ferraro and Miranda 2017). They are also straightforward to implement (as demonstrated in Supporting Information S7). Thus, we believe they are a key advance worth consideration by ecologists.

We illustrate how each design works using a common set of terms for a causal variable of interest (*x*; e.g. local temperature), the outcome or response variable (*y*; e.g. snail counts), and confounding variables (*w*; e.g. recruitment) in a regression. We consider data from different sites (*i*) sampled either at multiple time points in panel design or in multiple plots (*j*) in the case of a cross‐sectional data design as above. For the sake of simplicity, we assume a linear model form with normally distributed error (ϵ), although the framework also applies for generalised linear models. The model takes the form of

(1) $$y_{\mathrm{ij}}=\beta_{0}+\beta_{1}x_{\mathrm{ij}}+\gamma w_{i}+\epsilon_{\mathrm{ij}}$$

where *y*_*ij*_ is the abundance of snails at site *i* in year or plot *j*, $\beta_{0}$ is the intercept—the abundance of snails if the temperature and recruitment were 0, $\beta_{1}$ is the effect of temperature *x*_*ij*_ at site *i* in year or plot *j* on snails, γ is the effect of recruitment *w*_*i*_ at site *i* on snail abundance, and $\epsilon_{\mathrm{ij}}$ is other driver of snail abundance. Due to shared oceanographic influences, *x*_*ij*_ and *w*_*i*_ are correlated. *Thus, we have a confounding variable that varies spatially, across sites, and is correlated with average site temperature affecting snail abundance*. Our goal is to estimate $\beta_{1}$ (the effect of temperature on snail abundance) and eliminate the effect of confounding variables (and resulting bias). If we had measured *w*_*i*_, we could include it in a model, and by conditioning *w*, identify the effect of $\beta_{1}$ assuming no other confounders. Table S1 explains coefficient definitions for all models.

Without measuring and controlling for the confounder, *w*_*i*_, as above, we would instead fit the following naïve regression with only the abundance of snails (*y*_*ij*_) as a function of temperature (*x*_*ij*_) and random error:

(2) $$y_{\mathrm{ij}}=\beta_{0}+\beta_{1}x_{\mathrm{ij}}+\epsilon_{\mathrm{ij}}$$

Here, temperature (*x*_*ij*_) is endogenous—correlated to the error term; $w_{i}$ is implicitly included in the error term, creating omitted variable bias. Due to omitted variable bias, our causal inference about $\beta_{1}$ would be incorrect: different from the true causal effect.

### 6.1 What Ecologists Typically Do: Random or Mixed Effects Models That Fail to Solve OVB

Mixed effect models have been popular in Ecology for the past two decades (for useful reviews, see Bolker et al. 2009; Schielzeth and Nakagawa 2012; Harrison et al. 2018). Originally used to partition variation in heritability between different relatives (Fisher 1919), **random effects**—the effects of clusters in data assumed to come from a random distribution [but see Gelman and Hill (2006) on the linguistic ambiguities surrounding fixed and random effects]—quickly became a mainstay in the partitioning of variation in randomised experiments with subsamples taken within clusters (Cochran 1937; Eisenhart 1947). They have become a standard part of the toolbox for analysing ecological experiments (Schielzeth and Nakagawa 2012) and are frequently used when analysing observational data in Ecology.

Random effects account for clustering in data via the error structure of the model (Bolker et al. 2009; Gelman and Hill 2006), rather than estimating cluster means as part of the data generating process of a model (i.e., via fixed effect for each cluster's mean, using the terminology of the mixed models literature). This results in gains in efficiency (i.e., costing fewer degrees of freedom). As random effects are assumed to be drawn from a common distribution, they also have benefits for analyses of unbalanced samples and for regularising cluster means (i.e., shrinkage, drawing them towards the grand mean, see Efron and Morris 1975).

For these reasons, ecologists conducting a study akin to our snail‐temperature example could gravitate towards a mixed effect model to account for variation between sites in snail abundances, using a mixed effects model such as:

(3) $$\begin{array}{c} y_{\mathrm{ij}}=\beta_{0}+\beta_{1}x_{\mathrm{ij}}+\delta_{i}+\epsilon_{\mathrm{ij}}\\ \delta_{i}∼N\left(0,\sigma_{\text{site}}^{2}\right)\\ \epsilon_{\mathrm{ij}}∼N\left(0,\sigma^{2}\right) \end{array}$$

All coefficients are as in Equation (2) (see also Table S1), with the addition of $\delta_{i}$, the random effect—a site‐specific deviation of site *i* from the common intercept, $\beta_{0}$, due to variation assumed to follow a normal distribution with a mean of zero and variance of $\sigma_{\text{site}}^{2}$. However, if site is both a random effect and correlated with temperature, we cannot resolve the problem of OVB with this design.

### 6.2 What Assumptions Are Random Effect Design Making When It Comes to Omitted Variable Bias?

Why do random effect designs suffer from omitted variable bias with the above model not controlling for omitted confounders via its site random effect? To understand this problem, remember that, when using random effects, we are not estimating the effects of group means per se (Robinson 1991). Rather, we are modelling correlation in our error structure based on groups (Bolker et al. 2009; Schielzeth and Nakagawa 2012; Wooldridge 2010). This difference results in several efficiency gains and benefits discussed above but requires stricter assumptions for causal interpretation of estimated effects that are not often considered. The **Random Effects Assumption**—a variation on the assumption of no endogeneity—states that for the random effects estimator to be unbiased, the random effects, which are part of the error term, must not be correlated with any covariates in the regression (Antonakis, Bastardoz, and Rönkkö 2021; Wooldridge 2010). Using a mixed model for the snail data assumes that the random effect of site of is uncorrelated with temperature (Schielzeth and Nakagawa 2012; Wooldridge 2010). Given our DAG, we know this assumption is false; the model will not be causally identified and estimates of $\beta_{1}$ will be biased. Figure 3 shows this violation of the random effects assumption graphically with a path diagram for a model with the random effect model in Figure 3B,C. We posit that violations of the random effect assumption are likely common in Ecology—particularly in observational data that spans environmental gradients. The frequency and consequences of violating this assumption for Ecology is neither well explored nor acknowledged widely enough. We need solutions that require weaker assumptions that are less easily violated.

### 6.3 Enter the Econometric Fixed Effects Design

The Econometric Fixed Effects Design represents a familiar starting point for many ecologists used to using categorical variables in ANOVA and ANCOVA (e.g., Gotelli and Ellison 2012). Unfortunately, there are many uses of the term “fixed effect,” leading to a wealth of confusion across fields (Gelman and Hill 2006). Here, we use the term “fixed effect” as drawn from the econometrics literature, where it refers to attributes of a system (e.g., site, plot, or year) that vary by cluster (i.e., a within cluster intercept) that are encoded in models as dummy or categorical variables (e.g., representing sites or other descriptors of how our data is clustered). We also use “fixed effect” in the language of the mixed model literature—that is, that the cluster means are estimated as part of the data generating process of the model, not as part of the random error component.

Recognising that confounding variables vary at the cluster level (e.g., site), we have three options to flexibly control for the effects of confounding variables. First, we can use a bit of algebra known as the **within transformation** or **fixed effects estimator** (Bell, Fairbrother, and Jones 2018; Wooldridge 2010) that is similar to within‐subjects centering in Ecology (van de Pol and Wright 2009). To illustrate, we manipulate the following equation:

(4) $$y_{\mathrm{ij}}=\beta_{0}+\beta_{1}x_{\mathrm{ij}}+\epsilon_{\mathrm{ij}}+u_{i}$$

following notation of Equation (1), where $x_{\mathrm{ij}}$ is our casual variable of interest, but the error term is composed of idiosyncratic (random error), $\epsilon_{\mathrm{ij}}$, and $u_{i}$, which represent differences across sites *i* including unmeasured confounding variables (see Table S1). As site‐level confounders do not vary across within‐site replicates, $u_{i}=\bar{u_{i}}$. We can use this to our advantage via the fixed effect transformation. For this transformation, we remove the effect of site‐level confounding drivers, by subtracting the site‐level average value of $y–\bar{y_{i}}$–from both sides of the equation. On the right‐hand side, we can average over all terms at the site level to subtract $\beta_{0}+\beta_{1}\bar{x_{i}}+\bar{\epsilon_{i}}+\bar{u_{i}}$ which leads to a transformed model.

(5) $$\begin{array}{c} y_{\mathrm{ij}}-\bar{y_{i}}\;=\;\beta_{1}\left(x_{\mathrm{ij}}-\bar{x_{i}}\right)+\left(\epsilon_{\mathrm{ij}}-\bar{\epsilon_{i}}\right)+\left(u_{i}-\bar{u_{i}}\right)\\ \;=\;\beta_{1}\left(x_{\mathrm{ij}}-\bar{x_{i}}\right)+\left(\epsilon_{\mathrm{ij}}-\bar{\epsilon_{i}}\right) \end{array}$$

We have algebraically removed the confounding influence of time‐invariant site‐level confounding variables contained in $u_{i}$—whether they were observed or not.

**FIGURE 5:**
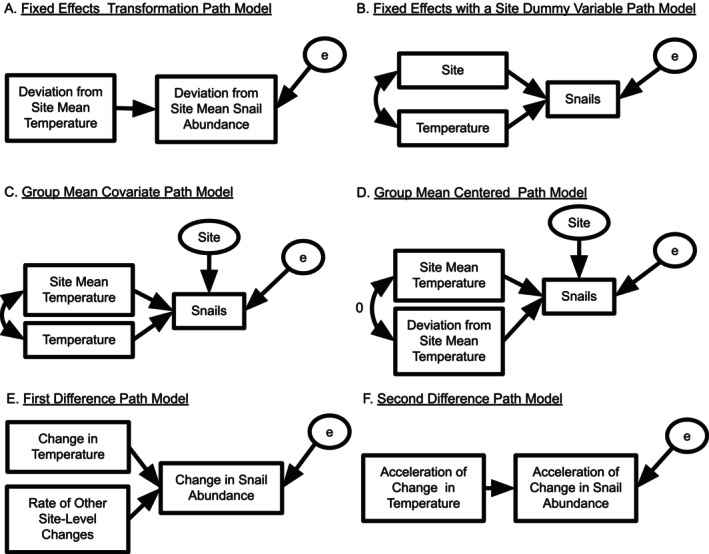
Path diagrams of different statistical models handling omitted variables in the text. (A) and (B) show two variations on the econometric fixed effect model design corresponding to Equations (5) and (6) respectively. (C) represents the group mean covariate design in Equation (7) and (D) represents the group mean centered design from Equation (8). Finally, (E) shows the first differencing approach from Equation (9) and (F) the second differencing approach. As the true error variables are unobserved, we include them in an ellipse.

Second, to achieve the same effect as this fixed effect transformation (see Figure 5A for a path diagram of the model), we could use a dummy variable for each cluster (i.e., a 0/1 encoding of *x*_2*i*_ for each cluster, an econometric fixed effect) multiplied by the cluster mean, $\lambda_{i}$, or simplify the equation to just use cluster means, $\lambda_{i}$, as a categorical variable per site as in Figure 5B. This is merely two ways of writing the same model.

(6) $$\begin{array}{c} y_{\mathrm{ij}}=\beta_{1}x_{1\mathrm{ij}}+\sum \lambda_{i}x_{2i}+\epsilon_{\mathrm{ij}}\\ \;=\beta_{1}x_{1\mathrm{ij}}+\lambda_{i}+\epsilon_{\mathrm{ij}} \end{array}$$

Unlike random effects in a mixed model design, $\lambda_{i}$ is not constrained to be drawn from a predefined probability distribution. These designs will control for omitted variable bias from site‐level observed and unobserved confounding variables and produce identical results to the preceding model for $\beta_{1}$ (Angrist and Pischke 2008; Wooldridge 2010), which we demonstrate with simulations below. While these two versions of the fixed effect design look different, they are equivalent (Angrist and Pischke 2008; Wooldridge 2010).

Fixed effect designs allow us to relax the strong assumption that all confounding variables are observed, measured, and included as covariates in models for a causal interpretation of $\beta_{1}$ when other assumptions are met (see Section 5). For ecological examples, see Larsen (2013), Dee et al. (2016, 2023), Dudney et al. (2021), and Ratcliffe et al. (2023).

**FIGURE 6:**
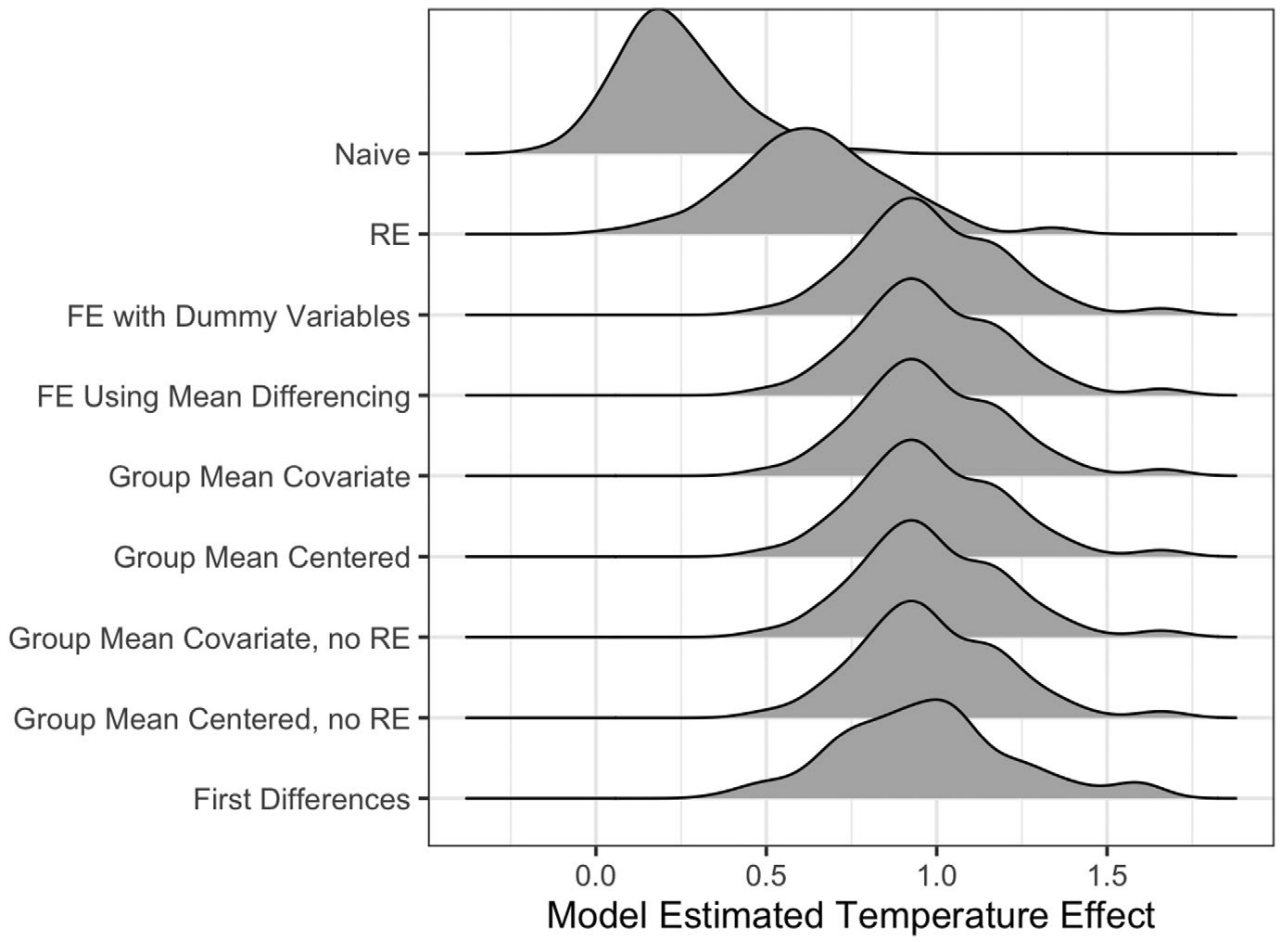
Distribution of point estimates of temperature effects from different models across all 100 simulations. The true effect size (=1) is highlighted with a dotted line. The y‐axis labels correspond to the Naïve model in Equation (2), Random Effects (RE) model in Equation (3), the Fixed Effects (FE) models in Equations (5) and (6), the Group Mean Covariate models in Equation (7) and the Group Mean Centered models to Equation (8), and the First Differences model in the Equation (9). The Naïve and Random Effects models produce biased coefficient estimates on average, in contrast to all other methods.

Fixed effect designs have some drawbacks despite their simplicity and strength in controlling for both observed and unobserved confounding variables. First, while these estimators make much weaker assumptions about confounding variables, they are inefficient compared to random effects. For each fixed effect (e.g., site), we estimate a separate coefficient. Estimating more parameters eats up degrees of freedom, requiring a larger sample size to achieve the same level of precision as random effects. This phenomenon creates a bias‐variance trade‐off (Bell, Fairbrother, and Jones 2018). If one's goal is causal inference, minimising bias is critical; thus, fixed effect designs are preferable over a mixed‐effects model (see simulation results in Table 1 and Figure 6). Finally, with the fixed effect approach, we lose information about between‐site variation, including gradients between sites that may be of interest. The fixed effects absorb this variation. These gradients, while confounded with other variables, could be the focus of some research questions that cannot be easily addressed using fixed effect designs.

### 6.4 Group Means for Efficiency, Inference, Fun, and Profit

To study between‐site variation and mitigate the loss of efficiency from the fixed effect design, we can instead use **correlated random effects** designs (using terminology of Antonakis, Bastardoz, and Rönkkö 2021). Correlated random effect designs use group means of our causal variable of interest to control for confounding variables. For each cluster (e.g., each site, year), researchers calculate a group mean of the causal variable of interest (e.g., average temperature of a site) and include it as a group‐level predictor. These group means of the causal variable control for the effects of confounders at the cluster level by acting as a proxy for confounders.

One correlated random effect design is the **Mundlak** **Device** (Mundlak 1978) and has many extensions (e.g., Wooldridge 2021). For clarity, we term it a **Group Mean Covariate** design, as in the following equation:

(7) $$\begin{array}{c} y_{\mathrm{ij}}=\beta_{0}+\beta_{1}x_{\mathrm{ij}}+\beta_{2}\bar{x_{i}}+\delta_{i}+\epsilon_{\mathrm{ij}}\\ \delta_{i}∼N\left(0,\sigma_{\text{site}}^{2}\right)\\ \;\epsilon_{\mathrm{ij}}∼N\left(0,\sigma^{2}\right) \end{array}$$

where $\beta_{2}\bar{x_{i}}$ with $\bar{x_{i}}$ as the average site temperature accounts for the effect of cluster‐level confounders and $\delta_{i}$ is a random effect of that cluster (i.e., site, other coefficients are as before or see Table S1). From the path model in Figure 5C, we can see the site mean temperature is statistically controlled in estimating the within‐site temperature effect.

Using group means of our causal variable enables us to estimate a coefficient for between‐cluster effects (e.g., between sites, $\beta_{2}$) called a **contextual effect** (Antonakis, Bastardoz, and Rönkkö 2021). These coefficients contain a combination of causal and confounded effects and are not causally identified. They should not be taken as strong evidence supporting or refuting a particular hypothesis. The coefficient for the contextual effect, here site mean temperature, quantifies how changing the mean temperature of a site—and all properties that correlate with site mean temperature—affects snail abundance if temperature within a plot stayed the same. For example, *if our plot was 10°C, what would snail abundance be if that plot was in a site with an average temperature of 5* versus *20°C*? If the contextual effect is 0, we can conclude that a simple mixed‐effect model would suffice and omitted variable bias was not substantial in this particular analysis (Antonakis, Bastardoz, and Rönkkö 2021).

The group mean covariate design will run into problems, however, if the correlation between our causal variable of interest and its cluster‐level mean is too high. To overcome this issue, we can instead use a **Group Mean Centering** design, which transforms our causal variable to remove this correlation. Group mean centering subtracts the cluster‐level mean from the causal variable of interest: $\left(x_{\mathrm{ij}}-\bar{x_{i}}\right).$ In our example, we subtract the site's average temperature across the whole time series from the observed temperature for each year at each site (see Figure 5D). After this transformation, we use this cluster‐level centered variable (i.e., within cluster anomaly) as our predictor variable of interest with $\beta_{1}$ estimating the effect of a 1° change in anomaly. We control for cluster level mean—which includes confounding effects—as follows:

(8) $$y_{\mathrm{ij}}=\beta_{0}+\beta_{1}\left(x_{\mathrm{ij}}-\bar{x_{i}}\right)+\beta_{2}\bar{x_{i}}+\delta_{i}+\epsilon_{\mathrm{ij}}$$

Equation (8) decomposes our causal variable of interest into between‐ and within‐cluster terms, $\beta_{2}$ and $\beta_{1}$, respectively. This is an approach already in use in Ecology (van de Pol and Wright 2009). The coefficient of the site mean temperature, $\beta_{2}$, is now the between‐site effect of temperature and confounders. The coefficient, $\beta_{1}$, is the within‐site temperature effect and is the same as previous models (Table S1). The interpretation of $\beta_{2}$ is different than in the group mean covariate design. $\beta_{2}$ for our snail example is a **between estimator** of the combined effect of moving across gradients in temperature and correlated drivers between the sites while holding anomaly constant. For example, if we moved from a site with a 5°C average temperature to one that was 10°C, how would snail abundance change in a plot holding anomaly constant? If the anomaly was 1°C, this would be a comparison between plots that were 6° at one site and 11°C at another. However, the result could be very different than comparing plots that were 6° and 11°C from the same site. If $\beta_{2}$ = $\beta_{1}$, omitted variables are not meaningfully influencing snail abundances; both the between and within site differences are due solely to temperature or multiple confounders have cancelled one another out.

The group mean covariate, group mean centered, and fixed effects designs all differ in structure but will yield the same point estimates of $\beta_{1}$ (Figure 6, Table 1) under most conditions and with balanced data (see simulations below and Wooldridge 2010). Mundlak (1978) showed that correlated random effects and fixed effects are algebraically identical in linear models and only differ in their inferences. Thus, one might ask: *which design should I use*? This decision depends on the structure and size of one's data (e.g., how many coefficients do you have the power to estimate given your sample size) and the question of interest (e.g., are you interested in between‐site differences?). For example, do you have many sites and are only interested in the causal effect of temperature? Fixed effects design. Do you want to know how plot‐level snail abundance would change if the average site temperature changes, but plot temperature stays the same? Group mean covariate design. Do you want to understand the effects of temperature while examining the net effect of many variables shaping between‐site gradients? Group mean centered design. Each design can further be extended to cases where the magnitude of the causal variable of interest's effect is moderated by the level of confounding variables (i.e., an interaction between unobserved confounders and our causal variable of interest—see Supporting Information S3: A Difficult Slope: Omitted Variables that Cause Variation in the Magnitude of the Causal Effect).

**TABLE 1:** Summary of simulation results comparing estimates from each study design compared to a naïve bivariate correlation.

Model type	Mean estimate	SD estimate	Fraction sims where 95% CI contains 0	Fraction sims where 95% CI does not contain 1
Naïve	0.231	0.165	0.56	0.99
Random effects (RE)	0.640	0.232	0.08	0.54
FE using mean differencing	0.985	0.215	0.00	0.05
FE with dummy variables	0.985	0.215	0.00	0.05
Group mean covariate	0.985	0.215	0.00	0.05
Group mean centered	0.985	0.215	0.00	0.05
Group mean covariate, no RE	0.985	0.215	0.01	0.04
Group mean centered, no RE	0.985	0.215	0.01	0.04
First differences	0.971	0.259	0.01	0.12

*Note:* Mean and SD of point estimates of temperature effects from different models in the first two columns. Fraction of simulated runs where the mean ± 2 SE of the temperature effect either overlapped 0 (i.e., high likelihood of committing a type II error) or did not contain the true effect of temperature in the final columns. Models are as in Figure 6.

Abbreviation: FE, econometric fixed effects.

Finally, for all of these designs, we note that accounting for serial correlation, heteroskedasticity, and clustering of the error through cluster robust standard errors or random effects at the cluster level are important for standard error estimation and thus inferences (Abadie et al. 2017; Cameron and Miller 2015). For more, see Supporting Information S4.

### 6.5 What a Difference Differencing Makes

Our examples thus far have focused on unobserved confounding variables that vary across space (i.e., between sites) rather than time. Time can be difficult, as it can enter the picture in several different ways. In the case of omitted confounders varying solely across time and not space, we can extend the frameworks presented above, using years rather than sites as clusters. If time‐varying confounders are uniform across sites, then we can use an econometric fixed effect of time and an econometric fixed effect of space (a two‐way fixed effect a.k.a. TWFE, Wooldridge 2021) and extensions (Roth et al. 2023) or a site‐average of predictors and a time‐average of predictors (a two‐way Mundlak model design; Wooldridge 2021).

If, however, temporal confounders differ by site, we need a more general solution. If our causal variable of interest has the same trend over time as temporal confounders, we can use **temporal differencing**. For example, consider ocean warming alongside coastal development at our sites (Figure S2) with development increasing over time, like warming, but varying in rate between sites. To separate these correlated drivers, we can use a **first‐difference design** (Figure 5e). We subtract all drivers and responses in year j from their value in year *j* − 1 (i.e., $\Delta y_{\mathrm{ij}}$ is change in snail abundance at site *i* between year *j* and *j* − 1). We illustrate this showing the subtraction to produce the first differences model:

(9) $$\begin{array}{c} y_{\mathrm{ij}}-y_{i\left(j-1\right)}=\beta_{1}x_{1\mathrm{ij}}-\beta_{1}x_{1i\left(j-1\right)}+\lambda_{i}j-\lambda_{i}\left(j-1\right)+\epsilon_{\mathrm{ij}}-\epsilon_{i\left(j-1\right)}\\ \Delta y_{\mathrm{ij}}=\beta_{1}\Delta x_{1\mathrm{ij}}+\lambda_{i}+\Delta \epsilon_{\mathrm{ij}} \end{array}$$

Here, $\lambda_{i}$ is the slope of the site‐specific temporal confounder. Spatial confounders are eliminated algebraically, just as in the fixed effect transformation. Finally, if we are uninterested in site‐specific trends, we can calculate the second difference $\Delta^{2}y_{\mathrm{ij}}=\Delta y_{\mathrm{ij}}-\Delta y_{i,j-1}$ eliminating the need to estimate $\lambda_{i}$ (Figure 5f). While both designs control for temporal and spatial confounding, they cost 1–2 years of data. Finally, if omitted confounders vary spatiotemporally without trends or spatial and temporal confounders interact, we can extend the principles discussed here to more exotic designs (see Box 2).

BOX 2 **Reality bites: coping with spatiotemporal omitted confounders.**

Spatiotemporal confounding variables—those that are site (or plot) specific and vary through time—pose challenges, and the solutions require more thoughtful study designs. To illustrate, we consider a scenario where recruitment, a confounding variable related to both snail abundance and temperature, is not static through time but instead varies by site and year. For example, sites that experience strong cold‐water pulses in a year also experience unusually high snail recruitment in those same years due to oceanographic drivers. The sampling designs for coping with spatiotemporal omitted variables are based on the same principles as cross‐sectional and longitudinal sampling, only now we combine the two to include plots within sites that are sampled through time.

Using longitudinal data with multiple plots sampled within a site through time, we can flexibly control for spatiotemporal confounding at the site level by extending the two‐way fixed effect designs discussed above. We can add a site‐by‐time fixed effect, $\eta_{\mathrm{ij}}$, to our model, in addition to a fixed effect of plot, $\lambda_{k}$, where *k* is a fixed plot within site resampled over time (see below for a discussion of fixed versus re‐randomised plots and Tables S1 and S2 for more on model terms and assumptions). This produces the following means model (i.e., not using dummy coding but just the means for the fixed effects):

$$y_{\mathrm{ijk}}=\beta_{1}x_{1\mathrm{ijk}}+\lambda_{k}+\eta_{\mathrm{ij}}+\epsilon_{\mathrm{ijk}}$$

From this equation, we can see that $\lambda_{k}$ captures time invariant plot‐level confounding effects while $\eta_{\mathrm{ij}}$ captures the effects of spatiotemporal omitted variables at the site by time level. See Table S1 for exact coefficient definitions. Note, there could be additional spatial or temporal only confounders. This design sweeps their effects onto the spatiotemporal term.

In small datasets, the above model design can consume degrees of freedom rapidly. In datasets with insufficient power, we can instead use the correlated random effects (e.g., a variation on the Two‐way Mundlak model design *sensu* Wooldridge 2021) which are more efficient. Correlated random effect use site‐year means ($\bar{x_{ij}}$) and plot means ($\bar{x_{k}}$) of temperature for the entire survey to control for spatiotemporal and plot confounding respectively:

$$y_{\mathrm{ijk}}=\beta_{0}+\beta_{1}x_{\mathrm{ijk}}+\beta_{2}\bar{x_{k}}+\beta_{3}\bar{x_{ij}}+\delta_{k}+\delta_{\mathrm{ij}}+\epsilon_{\mathrm{ijk}}$$

Here the $\delta_{k}$ and $\delta_{\mathrm{ij}}$ terms are random effects for plot and unique site‐time combinations respectively.

When sampling to handle spatiotemporal confounders, should plots within sites over time be permanent or randomly placed each year? The above models assume permanent plots, so we can eliminate confounding variables at the plot‐level that is time invariant over the study period. For this reason, permanent plots help us cope with within‐site OVB issues and have higher power to detect change over time (Urquhart and Kincaid 1999). Logistically, however, permanent plots within sites might not be possible. As such, the above models can be modified to drop plot effects; however, they would then assume that there are no confounding differences across plots and could have lower power to detect effects of drivers. We emphasise that the choice of fixed or random plot placement with these designs is a balancing act, however, as fixed plots can lead to a lower sample size due to logistical considerations in many environments, and direct readers to other explorations of this topic (e.g., Gomes 2022).

Finally, without variation within sites as well as through time—e.g., multiple plots sampled within sites each year—we cannot include a site by year effect as in the above models. We can attempt to use site‐level time trends (e.g., as linear or polynomial trends) as in Dee et al. (2016) or trends generated from Generalised Additive Models (Wood 2017) to approximate site‐by‐time effects, but this approach requires stronger assumptions about how confounders vary across sites over time. Therefore, we recommend researchers test the robustness of these assumptions. In cases without multiple plots per site sampled over time, researchers can use sensitivity tests (Oster 2019) or partial identification (Ghassami, Shpitser, and Tchetgen 2024; Miao, Geng, and Tchetgen Tchetgen 2018) to get the bounds of estimates. In situations where these bounds are wide, however, without multiple plots per site, then, “nothing to be done” (Beckett 1954).

In general, we urge caution when dealing with spatiotemporal omitted variables, and careful use of causal diagrams to ensure that we are controlling for confounders at the relevant spatiotemporal scale. This topic deserves far more exploration in Ecology.

## 7 Comparison of Approaches

To demonstrate the utility of the preceding solutions and the consequences of not using them, we fit a variety of models to simulated data based on a longitudinal study of snail populations at multiple sites based on Figure 2. Snail abundance at site *i* in year *j* is a function of recruitment, temperature, and other unobserved confounded drivers. For a single simulation run, we created a system as follows, simulating 10 sites over 10 years using a panel sampling design with:

- Oceanography as a random normal variable with a mean of 0 and standard deviation of 1. *O*_*i*_ ~ *N*(0, 1).
- Site mean recruitment calculated as −2 multiplied by the oceanography variable and then rescaled to have a mean of 10 individuals per plot (e.g., so it does not go negative). It is the same in a site across all years. *R*_*i*_ | *O*_*i*_ = −2 *O*_*i*_ + 10
- Site mean temperature as calculated as twice the oceanography variable and then rescaled to have a mean of 15C. *T*_*i*_ | *O*_*i*_ = 2 *O*_*i*_ + 15
- Site temperature in year *j* determined by site mean temperature and additional variation random normal variation with a mean of 0 and standard deviation of 1. *T*_*ij*_ ~ *N*(*T*_*i*_, 1)
- Snail abundance at site *i* in is determined in a given year as in Figure 2, as a function of recruitment, temperature, and other drivers; the effects of both temperature and recruitment on snail abundance are 1. Other drivers are drawn from a random normal distribution with a mean effect of 0 and standard deviation of 1. *S*_*ij*_ | (*T*_*ij*_, *R*_*i*_) ~ *N*(*T*_*ij*_ + *R*_*i*_, 1)

Simulation code and results from 100 simulated data sets are in Supporting Information S6 and at https://doi.org/10.5281/zenodo.13737990. We analysed each simulation run using all of the statistical designs above, including a naïve model with no site effect. We also included group mean covariate and group mean centered models with and without a random effect of site to demonstrate the role of a random effect in estimating standard errors and handling unbalanced data. Supporting Information S7 uses Supporting Data S1 to analyse one data set showing the simplicity of implementation. For an interactive exploration of the full suite of simulated data and parameters, see the web applications in Supporting Information S8 (for one simulated run alone) and Supporting Information S9 (using replicated simulations to explore parameter distributions).

Our simulations show that the RE model—what ecologists typically use—is consistently biased in these simulations. The point estimates from RE model are well‐below the estimates from both the other designs and the true effect size (Figure 6, Table 1). Not only is the estimated coefficient of the RE model always biased compared to other estimators in our simulations, but it is also more often within 2SE of 0 (i.e., would fail to reject a null hypothesis) in comparison to all other designs. More worrying, in the majority (54%) of simulations, the 95% confidence intervals of the RE model do not contain the true effect of 1 (Table 1). Other than the naïve and random effects model, the other designs show similar estimates with balanced data (in Table 1). The first differences model underperforms with respect to its CI not containing the true parameter value relative to other designs, but it performs far better than the RE and naïve design. However, the relative performance of first difference versus econometric fixed effect depends on the structure of data, whether it has more time periods, or more units (Wooldridge 2010), which likely explains this discrepancy.

For estimating standard errors, in general, we urge researchers to incorporate random effects or clustered robust standard errors as needed to accommodate clustering and heteroskedasticity in the error, recognising the tradeoffs of using both and appropriate context (reviewed in Oshchepkov and Shirokanova 2022). For instance, a site‐level random effect improves efficiency and precision for group mean centered or group mean covariate models when either the study design is unbalanced or there is site‐level variation that is uncorrelated with temperature (for more details, see Supporting Information S6). If our simulation has no site‐level variation other than temperature and our confounder, a random effect does not improve either models' ability to estimate the effect of our causal variable of interest with respect to bias or precision.

## 8 Discussion

We aim to introduce and spur the broader uptake of techniques addressing omitted variable bias for causal inference with observational data. At the core of these and other causal inference techniques is an *a priori* causal model of how a system works to guide sampling, statistical design choices, and to clarify assumptions required for a causal interpretation of estimated effects. Inferences made from designs that better control for unobserved confounding variables can improve our ability to understand biological systems, as seen in our simulations and results. Further, the techniques presented here are well within the standard statistical abilities of most modern ecologists (see Supporting Information S7 for R code).

We hope that ecologists can see the concepts presented here as part of a generalisable approach to handling confounding variables using clustered or hierarchical data. While we use sites and years, the same concepts apply to studies with cohort effects, individual effects, or other lower levels of clustering as well as larger‐scale studies with not just sites and years but regions and decades. Cross‐sectional and longitudinal sampling designs are also generalisable beyond our example's simple case, including for spatiotemporal designs (see Box 2). Combining these sampling designs with others, such as a stratified random sampling design (Foster et al. 2018; Grafström and Lundström 2013; Kermorvant et al. 2019; Robertson et al. 2013; Stevens and Olsen 2004) will allow for the analyses that can both improve causal identification and also provide more precision in estimation over multiple environmental gradients. How to design a study to fully account for confounders, however, will hinge on a causal structure of the system and a researcher's ability to be humble in the face of what they might not know.

The approaches presented here are not a panacea. They require some of the same assumptions as experiments (Kimmel et al. 2021) for causal inference, that is, the Stable Unit Treatment Value Assumption (SUTVA) (reviewed in Kimmel et al. 2021). They require additional assumptions as well regarding confounders (see Table S2). The statistical designs presented here include assumptions, at least on the link scale, that effects are linear, additive (Imai and Kim 2021), and homogeneous. We have included discussion of relaxing these assumptions via interactions (Supporting Information S3) and refer readers to a growing literature on estimating causal effects under heterogeneity and non‐linearity (Callaway and Sant'Anna 2021; de Chaisemartin and D'Haultfœuille 2020; Goodman‐Bacon 2021; Sun and Abraham 2021) or using flexible machine learning (Athey, Tibshirani, and Wager 2019; Athey and Imbens 2019; Fink et al. 2023). Generalised linear models can exhibit a slight downward bias for some distributions and link functions, although this appears largely negligible (Bell, Fairbrother, and Jones 2018; Brumback et al. 2010); techniques for consistent estimation are under active development (Schunck and Perales 2017).

The approaches presented here also make the parallel trend assumption. It implies that, without a driver, the *difference* in outcomes between different clusters is constant through time conditioned on covariates. The parallel trends assumption has come under scrutiny (reviewed in Roth et al. 2023) when changes in the causal variable of interest happen at different points in time across units (Baker, Larcker, and Wang 2022; Marcus and Sant'Anna 2021) or with heterogeneous effects (see Borusyak, Jaravel, and Spiess 2024; de Chaisemartin and D'Haultfœuille 2020; Goodman‐Bacon 2021; Sun and Abraham 2021). This assumption also holds true for before‐after‐control‐impact analyses (a.k.a. BACI, Difference‐in‐Differences, or Diff‐in‐Diff designs). Solutions are evolving rapidly, including for heterogeneous effects (see Roth et al. 2023), nonlinearities (Imai and Kim 2021), and continuous causal variables (Callaway, Goodman‐Bacon, and Sant'Anna 2024). They are already being implemented in software (reviewed in Roth et al. 2023).

The important thing is to be transparent in how we deal with assumptions required for causal interpretation of estimates. What are the assumptions you are making to interpret an effect as causal? Why did you control for some covariates and not others? Do you have a DAG or conceptual model of your system to help a reader understand your thought process? With mixed models, do you meet the random effects assumption? Are your clusters uncorrelated with your predictor of interest? Why or why not? Do you need to implement robust standard errors given your residual structure? Clarifying these and other decisions, even in a brief sentence if not a figure or full breakdown in a manuscript supplement (e.g., see Dee et al. 2023), will make analyses more transparent and allow others to build on your work to advance science. Further, in concert with the approaches presented here, we suggest using sensitivity tests (Altonji, Elder, and Taber 2005; Cinelli and Hazlett 2020; Oster 2019; Rosenbaum 2002) or designs that differ in assumptions to assess robustness of results [see Dee et al. (2023) for an ecological example]. At the end of the day, we must be humble and accept that our models and knowledge are imperfect. Someday, someone will come along with a different approach or new data that produces different conclusions and yields new insights. That is just part of the scientific process.

Finally, we emphasise that this paper provides an entry point into a broader, interdisciplinary literature on causal inference (see Supporting Information S5 for useful texts). Other quasi‐experimental designs, such as instrumental variables, synthetic control approaches, regression discontinuity designs, and more can also be used to eliminate omitted variable bias (see Arif and MacNeil 2022b; Butsic et al. 2017; Dee et al. 2023; Fick et al. 2021; Grace 2021; Kendall 2015; Larsen, Meng, and Kendall 2019; MacDonald and Mordecai 2019). Thoughtful uses of the front‐door criterion—using mediators between a cause and effect unaffected by confounders—could also prove useful for causal analysis (Bellemare, Bloem, and Wexler 2024; Pearl, Glymour, and Jewell 2016); although, there are no examples in the ecological literature yet to our knowledge. We urge ecologists, long grounded in experiments, to consider these and other transdisciplinary advances in causal inference in observational data as an important complement to experiments.

## 9 Conclusions

“Correlation does not equal causation” rings in many of our heads from Biostatistics 101. A key reason behind this message is the spectre of omitted variable bias. This fear has impeded the use of observational data for causal inference in Ecology for much of its recent history. We hope this review can lift some of that fear and, armed with the new tools and a knowledge of the literature beyond this piece (see above), we can move forward as a discipline. With a massively growing volume of observational data and problems at continental to global scales demanding rapid answers, we look forward to seeing ecologists harness these techniques to answering crucial questions in the future.

## Author Contributions

Authors contributed equally and share first authorship. **Jarrett E. K. Byrnes:** conceptualization, methodology, software, validation, formal analysis, writing – original draft, review, and editing, visualisation. **Laura E. Dee:** conceptualization, methodology, validation, formal analysis, writing – original draft, review, and editing, visualisation.

### Peer Review

The peer review history for this article is available at https://www.webofscience.com/api/gateway/wos/peer‐review/10.1111/ele.70023.

References

Abadie, A.
, 
S.
Athey
, 
G. W.
Imbens
, and 
J.
Wooldridge
. 2017. “When Should You Adjust Standard Errors for Clustering?” (Working Paper No. 24003). Working Paper Series. National Bureau of Economic Research.

Abdallah, W.
, 
M.
Goergen
, and 
N.
O'Sullivan
. 2015. “Endogeneity: How Failure to Correct for It Can Cause Wrong Inferences and Some Remedies.” British Journal of Management
26: 791–804.

Altonji, J. G.
, 
T. E.
Elder
, and 
C. R.
Taber
. 2005. “Selection on Observed and Unobserved Variables: Assessing the Effectiveness of Catholic Schools.” Journal of Political Economy
113: 151–184.

Angrist, J. D.
, and 
J.‐S.
Pischke
. 2008. Mostly Harmless Econometrics. Princeton: Princeton University Press.

Antonakis, J.
, 
N.
Bastardoz
, and 
M.
Rönkkö
. 2021. “On Ignoring the Random Effects Assumption in Multilevel Models: Review, Critique, and Recommendations.” Organizational Research Methods
24: 443–483.

Antonakis, J.
, 
S.
Bendahan
, 
P.
Jacquart
, and 
R.
Lalive
. 2010. “On Making Causal Claims: A Review and Recommendations.” Leadership Quarterly Yearly Review
21: 1086–1120.

Arif, S.
, and 
M. A.
MacNeil
. 2022a. “Predictive Models Aren't for Causal Inference.” Ecology Letters
25: 1741–1745.35672133
10.1111/ele.14033

Arif, S.
, and 
M. A.
MacNeil
. 2022b. “Utilizing Causal Diagrams Across Quasi‐Experimental Approaches.” Ecosphere
13: e4009.

Arif, S.
, and 
M. A.
MacNeil
. 2023. “Applying the Structural Causal Model Framework for Observational Causal Inference in Ecology.” Ecological Monographs
93: e1554.

Athey, S.
, and 
G. W.
Imbens
. 2019. “Machine Learning Methods That Economists Should Know About.” Annual Review of Economics
11: 685–725.

Athey, S.
, 
J.
Tibshirani
, and 
S.
Wager
. 2019. “Generalized Random Forests.” Annals of Statistics
47: 1148–1178.

Baker, A.
, 
D. F.
Larcker
, and 
C. C. Y.
Wang
. 2022. “How Much Should We Trust Staggered Difference‐In‐Differences Estimates?” Journal of Financial Economics
144: 370–395.

Beckett, S.

1954. Waiting for Godot: Tragicomedy in 2 Acts. Evergreen Book. New York: Grove Press.

Bell, A.
, 
M.
Fairbrother
, and 
K.
Jones
. 2018. “Fixed and Random Effects Models: Making an Informed Choice.” Quality and Quantity
55: 117.

Bellemare, M. F.
, 
J. R.
Bloem
, and 
N.
Wexler
. 2024. “The Paper of How: Estimating Treatment Effects Using the Front‐Door Criterion.” Oxford Bulletin of Economics and Statistics
86: 951–993.

Bolker, B. M.
, 
M. E.
Brooks
, 
C. J.
Clark
, et al. 2009. “Generalized Linear Mixed Models: A Practical Guide for Ecology and Evolution.” Trends in Ecology & Evolution
24: 127–135.19185386
10.1016/j.tree.2008.10.008

Borusyak, K.
, 
X.
Jaravel
, and 
J.
Spiess
. 2024. “Revisiting Event Study Designs: Robust and Efficient Estimation.” Review of Economic Studies
rdae007: 3253–3285.

Broitman, B. R.
, 
C. A.
Blanchette
, and 
S. D.
Gaines
. 2005. “Recruitment of Intertidal Invertebrates and Oceanographic Variability at Santa Cruz Island, California.” Limnology and Oceanography
50: 1473–1479.

Brumback, B. A.
, 
A. B.
Dailey
, 
L. C.
Brumback
, 
M. D.
Livingston
, and 
Z.
He
. 2010. “Adjusting for Confounding by Cluster Using Generalized Linear Mixed Models.” Statistics & Probability Letters
80: 1650–1654.

Butsic, V.
, 
D. J.
Lewis
, 
V. C.
Radeloff
, 
M.
Baumann
, and 
T.
Kuemmerle
. 2017. “Quasi‐Experimental Methods Enable Stronger Inferences From Observational Data in Ecology.” Basic and Applied Ecology
19: 1–10.

Callaway, B.
, 
A.
Goodman‐Bacon
, and 
P. H. C.
Sant'Anna
. 2024. “Difference‐In‐Differences With a Continuous Treatment.” (Working Paper No. 32117). Working Paper Series. National Bureau of Economic Research.

Callaway, B.
, and 
P. H. C.
Sant'Anna
. 2021. “Difference‐In‐Differences With Multiple Time Periods.” Journal of Econometrics
225: 200–230.

Cameron, A. C.
, and 
D. L.
Miller
. 2015. “A Practitioner's Guide to Cluster‐Robust Inference.” Journal of Human Resources
50: 317–372.

Cinelli, C.
, and 
C.
Hazlett
. 2020. “Making Sense of Sensitivity: Extending Omitted Variable Bias.” Journal of the Royal Statistical Society Series B: Statistical Methodology
82: 39–67.

Cochran, W. G.

1937. “Problems Arising in the Analysis of a Series of Similar Experiments.” Supplement to the Journal of the Royal Statistical Society
4: 102–118.

de Chaisemartin, C.
, and 
X.
D'Haultfœuille
. 2020. “Two‐Way Fixed Effects Estimators With Heterogeneous Treatment Effects.” American Economic Review
110: 2964–2996.

Dee, L. E.
, 
P. J.
Ferraro
, 
C. N.
Severen
, et al. 2023. “Clarifying the Effect of Biodiversity on Productivity in Natural Ecosystems With Longitudinal Data and Methods for Causal Inference.” Nature Communications
14: 2607.10.1038/s41467-023-37194-5PMC1016323037147282

Dee, L. E.
, 
S. J.
Miller
, 
L. E.
Peavey
, et al. 2016. “Functional Diversity of Catch Mitigates Negative Effects of Temperature Variability on Fisheries Yields.” Proceedings of the Royal Society B: Biological Sciences
283: 20161435.10.1098/rspb.2016.1435PMC501377627534960

Dudney, J.
, 
C. E.
Willing
, 
A. J.
Das
, 
A. M.
Latimer
, 
J. C. B.
Nesmith
, and 
J. J.
Battles
. 2021. “Nonlinear Shifts in Infectious Rust Disease due to Climate Change.” Nature Communications
12: 5102.10.1038/s41467-021-25182-6PMC838505134429405

Efron, B.
, and 
C.
Morris
. 1975. “Data Analysis Using Stein's Estimator and Its Generalizations.” Journal of the American Statistical Association
70: 311–319.

Eisenhart, C.

1947. “The Assumptions Underlying the Analysis of Variance.” Biometrics
3: 1–21.20240414


Ferraro, P. J.
, and 
J. J.
Miranda
. 2017. “Panel Data Designs and Estimators as Substitutes for Randomized Controlled Trials in the Evaluation of Public Programs.” Journal of the Association of Environmental and Resource Economists
4: 281–317.

Fick, S. E.
, 
T. W.
Nauman
, 
C. C.
Brungard
, and 
M. C.
Duniway
. 2021. “Evaluating Natural Experiments in Ecology: Using Synthetic Controls in Assessments of Remotely Sensed Land Treatments.” Ecological Applications
31: e02264.33220145
10.1002/eap.2264

Fink, D.
, 
A.
Johnston
, 
M.
Strimas‐Mackey
, et al. 2023. “A Double Machine Learning Trend Model for Citizen Science Data.” Methods in Ecology and Evolution
14: 2435–2448.

Fisher, R. A.

1919. “XV. The Correlation Between Relatives on the Supposition of Mendelian Inheritance.” Earth and Environmental Science Transactions of the Royal Society of Edinburgh
52: 399–433.

Foster, S.
, 
J.
Monk
, 
E.
Lawrence
, 
K.
Hayes
, 
G.
Hosack
, and 
R.
Przeslawski
. 2018. “Statistical Considerations for Monitoring and Sampling.” In Field Manuals for Marine Sampling to Monitor Australian Waters, edited by 

R.
Przeslawski

 and 

S.
Foster

, 23–41. National Environmental Science Programme (NESP).

Gelman, A.
, and 
J.
Hill
. 2006. Data Analysis Using Regression and Multilevel/Hierarchical Models. Cambridge: Cambridge University Press.

Ghassami, A.
, 
I.
Shpitser
, and 
E. T.
Tchetgen
. 2024. “Partial Identification of Causal Effects Using Proxy Variables.”
*arXiv*. 10.48550/arXiv.2304.04374.

Glymour, C.
, 
K.
Zhang
, and 
P.
Spirtes
. 2019. “Review of Causal Discovery Methods Based on Graphical Models.” Frontiers in Genetics
10: 524.31214249
10.3389/fgene.2019.00524PMC6558187

Gomes, D. G. E.

2022. “Should I Use Fixed Effects or Random Effects When I Have Fewer Than Five Levels of a Grouping Factor in a Mixed‐Effects Model?” PeerJ
10: e12794.35116198
10.7717/peerj.12794PMC8784019

Goodman‐Bacon, A.

2021. “Difference‐In‐Differences With Variation in Treatment Timing.” Journal of Econometrics
225: 254–277.

Gotelli, N. J.
, and 
A. M.
Ellison
. 2012. A Primer of Ecological Statistics. 2nd ed. Oxford, New York: Oxford University Press.

Grace, J. B.

2021. “Instrumental Variable Methods in Structural Equation Models.” Methods in Ecology and Evolution
12: 1148–1157.

Grace, J. B.
, and 
K. M.
Irvine
. 2020. “Scientist's Guide to Developing Explanatory Statistical Models Using Causal Analysis Principles.” Ecology
101: e02962.31872426
10.1002/ecy.2962

Grafström, A.
, and 
N.
Lundström
. 2013. “Why Well Spread Probability Samples Are Balanced.” Open Journal of Statistics
3: 36–41.

Griffith, G. J.
, 
T. T.
Morris
, 
M. J.
Tudball
, et al. 2020. “Collider Bias Undermines Our Understanding of COVID‐19 Disease Risk and Severity.” Nature Communications
11: 5749.10.1038/s41467-020-19478-2PMC766502833184277

Harrison, X. A.
, 
L.
Donaldson
, 
M. E.
Correa‐Cano
, et al. 2018. “A Brief Introduction to Mixed Effects Modelling and Multi‐Model Inference in Ecology.” PeerJ
6: e4794.29844961
10.7717/peerj.4794PMC5970551

Heckman, J. J.

2000. “Causal Parameters and Policy Analysis in Economics: A Twentieth Century Retrospective*.” Quarterly Journal of Economics
115: 45–97.

Hernan, M. A.
, and 
J. M.
Robins
. 2023. Causal Inference: What If. Boca Raton: CRC Press.

Holland, P. W.

1986. “Statistics and Causal Inference.” Journal of the American Statistical Association
81: 945–960.

Imai, K.
, and 
I. S.
Kim
. 2021. “On the Use of Two‐Way Fixed Effects Regression Models for Causal Inference With Panel Data.” Political Analysis
29: 405–415.

Imbens, G. W.
, and 
D. B.
Rubin
. 2015. Causal Inference for Statistics, Social, and Biomedical Sciences: An Introduction. Cambridge: Cambridge University Press.

Kendall, B. E.

2015. “A Statistical Symphony: Instrumental Variables Reveal Causality and Control Measurement Error.” In Ecological Statistics: Contemporary Theory and Application, edited by 

G. A.
Fox

, 

S.
Negrete‐Yankelevich

, and 

V. J.
Sosa

. Oxford: Oxford University Press.

Kermorvant, C.
, 
F.
D'Amico
, 
N.
Bru
, 
N.
Caill‐Milly
, and 
B.
Robertson
. 2019. “Spatially Balanced Sampling Designs for Environmental Surveys.” Environmental Monitoring and Assessment
191: 524.31363924
10.1007/s10661-019-7666-y

Kimmel, K.
, 
L. E.
Dee
, 
M. L.
Avolio
, and 
P. J.
Ferraro
. 2021. “Causal Assumptions and Causal Inference in Ecological Experiments.” Trends in Ecology & Evolution
36: 1141–1152.34538502
10.1016/j.tree.2021.08.008

Larsen, A. E.

2013. “Agricultural Landscape Simplification Does Not Consistently Drive Insecticide Use.” Proceedings of the National Academy of Sciences
110: 15330–15335.10.1073/pnas.1301900110PMC378087824003135

Larsen, A. E.
, 
K.
Meng
, and 
B. E.
Kendall
. 2019. “Causal Analysis in Control–Impact Ecological Studies With Observational Data.” Methods in Ecology and Evolution
10: 924–934.

Laubach, Z. M.
, 
E. J.
Murray
, 
K. L.
Hoke
, 
R. J.
Safran
, and 
W.
Perng
. 2021. “A Biologist's Guide to Model Selection and Causal Inference.” Proceedings of the Royal Society B: Biological Sciences
288: 20202815.10.1098/rspb.2020.2815PMC789325533499782

MacDonald, A. J.
, and 
E. A.
Mordecai
. 2019. “Amazon Deforestation Drives Malaria Transmission, and Malaria Burden Reduces Forest Clearing.” Proceedings of the National Academy of Sciences
116: 22212–22218.10.1073/pnas.1905315116PMC682531631611369

Marcus, M.
, and 
P. H. C.
Sant'Anna
. 2021. “The Role of Parallel Trends in Event Study Settings: An Application to Environmental Economics.” Journal of the Association of Environmental and Resource Economists
8: 235–275.

McElreath, R.

2020. Statistical Rethinking: A Bayesian Course With Examples in R and Stan. Boca Raton: Chapman and Hall/CRC.

Miao, W.
, 
Z.
Geng
, and 
E. J.
Tchetgen Tchetgen
. 2018. “Identifying Causal Effects With Proxy Variables of an Unmeasured Confounder.” Biometrika
105: 987–993.33343006
10.1093/biomet/asy038PMC7746017

Morgan, S. L.
, and 
C.
Winship
. 2015. Counterfactuals and Causal Inference. Cambridge: Cambridge University Press.

Mundlak, Y.

1978. “On the Pooling of Time Series and Cross Section Data.” Econometrica
46: 69–85.

Oshchepkov, A.
, and 
A.
Shirokanova
. 2022. “Bridging the Gap Between Multilevel Modeling and Economic Methods.” Social Science Research
104: 102689.35400392
10.1016/j.ssresearch.2021.102689

Oster, E.

2019. “Unobservable Selection and Coefficient Stability: Theory and Evidence.” Journal of Business & Economic Statistics
37: 187–204.

Pearl, J.

1995. “Causal Diagrams for Empirical Research.” Biometrika
82: 669–688.

Pearl, J.

2009. Causality. Cambridge: Cambridge University Press.

Pearl, J.
, 
M.
Glymour
, and 
N. P.
Jewell
. 2016. Causal Inference in Statistics: A Primer. Hoboken: John Wiley & Sons.

Ratcliffe, H.
, 
A.
Kendig
, 
S.
Vacek
, 
D.
Carlson
, 
M.
Ahlering
, and 
L. E.
Dee
. 2023. “Extreme Precipitation Promotes Invasion in Managed Grasslands.” Ecology
105: e4190.37877294
10.1002/ecy.4190

Rinella, M. J.
, 
D. J.
Strong
, and 
L. T.
Vermeire
. 2020. “Omitted Variable Bias in Studies of Plant Interactions.” Ecology
101: e03020.32083313
10.1002/ecy.3020

Robertson, B. L.
, 
J. A.
Brown
, 
T.
McDonald
, and 
P.
Jaksons
. 2013. “BAS: Balanced Acceptance Sampling of Natural Resources.” Biometrics
69: 776–784.23844595
10.1111/biom.12059

Robins, J.

1989. “The Control of Confounding by Intermediate Variables.” Statistics in Medicine
8: 679–701.2749074
10.1002/sim.4780080608

Robinson, G. K.

1991. “That BLUP Is a Good Thing: The Estimation of Random Effects.” Statistical Science
6: 15–32.

Rosenbaum, P. R.

2002. Observational Studies. Springer Series in Statistics. New York, NY: Springer.

Roth, J.
, 
P. H. C.
Sant'Anna
, 
A.
Bilinski
, and 
J.
Poe
. 2023. “What's Trending in Difference‐In‐Differences? A Synthesis of the Recent Econometrics Literature.” Journal of Econometrics
235: 2218–2244.

Rubin, D. B.

1974. “Estimating Causal Effects of Treatments in Randomized and Nonrandomized Studies.” Journal of Education & Psychology
66: 688–701.

Rubin, D. B.

2005. “Causal Inference Using Potential Outcomes.” Journal of the American Statistical Association
100: 322–331.

Ruesink, J.

2000. “Intertidal Mesograzers in Field Microcosms: Linking Laboratory Feeding Rates to Community Dynamics.” Journal of Experimental Marine Biology and Ecology
248: 163–176.10771300
10.1016/s0022-0981(00)00170-2

Schennach, S. M.

2016. “Recent Advances in the Measurement Error Literature.” Annual Review of Economics
8: 341–377.

Schielzeth, H.
, and 
S.
Nakagawa
. 2012. “Nested by Design: Model Fitting and Interpretation in a Mixed Model Era.” Methods in Ecology and Evolution
4: 14–24.

Schunck, R.
, and 
F.
Perales
. 2017. “Within‐ and Between‐Cluster Effects in Generalized Linear Mixed Models: A Discussion of Approaches and the Xthybrid Command.” Stata Journal
17: 89–115.

Simler‐Williamson, A. B.
, and 
M. J.
Germino
. 2022. “Statistical Considerations of Nonrandom Treatment Applications Reveal Region‐Wide Benefits of Widespread Post‐Fire Restoration Action.” Nature Communications
13: 3472.10.1038/s41467-022-31102-zPMC920349835710763

Stachowicz, J. J.
, 
R. J.
Best
, 
M. E. S.
Bracken
, and 
M. H.
Graham
. 2008. “Complementarity in Marine Biodiversity Manipulations: Reconciling Divergent Evidence From Field and Mesocosm Experiments.” Proceedings of the National Academy of Sciences
105: 18842–18847.10.1073/pnas.0806425105PMC259622219028868

Stevens, D. L.
, and 
A. R.
Olsen
. 2004. “Spatially Balanced Sampling of Natural Resources.” Journal of the American Statistical Association
99: 262–278.

Sun, L.
, and 
S.
Abraham
. 2021. “Estimating Dynamic Treatment Effects in Event Studies With Heterogeneous Treatment Effects.” Journal of Econometrics
225: 175–199.

Textor, J.
, 
B.
van der Zander
, 
M. S.
Gilthorpe
, 
M.
Liśkiewicz
, and 
G. T.
Ellison
. 2016. “Robust Causal Inference Using Directed Acyclic Graphs: The R Package ‘Dagitty’.” International Journal of Epidemiology
45: 1887–1894.28089956
10.1093/ije/dyw341

Urquhart, N. S.
, and 
T. M.
Kincaid
. 1999. “Designs for Detecting Trend From Repeated Surveys of Ecological Resources.” Journal of Agricultural, Biological, and Environmental Statistics
4: 404–414.

van de Pol, M.
, and 
J.
Wright
. 2009. “A Simple Method for Distinguishing Within‐ Versus Between‐Subject Effects Using Mixed Models.” Animal Behaviour
77: 753–758.

Wolkovich, E. M.
, 
B. I.
Cook
, 
J. M.
Allen
, et al. 2012. “Warming Experiments Underpredict Plant Phenological Responses to Climate Change.” Nature
485: 494–497.22622576
10.1038/nature11014

Wood, S. N.

2017. Generalized Additive Models: An Introduction With R. 2nd ed. New York: Chapman and Hall/CRC.

Wooldridge, J. M.

2010. Econometric Analysis of Cross Section and Panel Data. Cambridge: MIT Press.

Wooldridge, J. M.

2015. Introductory Econometrics: A Modern Approach. Mason, Ohio: Cengage Learning.

Wooldridge, J. M.

2021. “Two‐Way Fixed Effects, the Two‐Way Mundlak Regression, and Difference‐In‐Differences Estimators.” Social Science Research Network 3906345.

Yund, P. O.
, 
C. E.
Tilburg
, and 
M. A.
McCartney
. 2015. “Across‐Shelf Distribution of Blue Mussel Larvae in the Northern Gulf of Maine: Consequences for Population Connectivity and a Species Range Boundary.” Royal Society Open Science
2: 150513.27018654
10.1098/rsos.150513PMC4807459

## Supporting information


**Table S1.** Models, equations, and the definition of coefficients based on linear regression models.
**Table S2.** Key assumptions, drawbacks, and types of Omitted Variable Bias handled by statistical model designs in this paper.
**Supporting Information S1**. Additional details and examples of Directed Acyclic Graphs and confounding.
**Figure S1.** Examples of statistical control for confounding variables informed by causal graphs.
**Supporting Information S2.** Directed Acyclic Graphs: What about feedbacks?
**Supporting Information S3.** A Difficult Slope: Omitted Variables that cause variation in the magnitude of the causal effect.
**Supporting Information S4.** Clustered Robust Standard Errors: An Underutilised Tool in Ecology.
**Figure S2.** Directed Acyclic Graph of a scenario with spatial and temporal omitted variables where site‐level temporal confounders are correlated with the causal variable of interest.
**Supporting Information S5.** Additional recommendations for readings.

**Supporting Information S6.** Models and Simulations to Evaluate the Consequences of Model Structure for Omitted Variable Bias.

**Supporting Information S7.** Implementing OVB Model Methods in R.

**Supporting Information S8.** Code for R Shiny App to simulate a data set and compare results of statistical designs over just that data set.

**Supporting Information S9.** Code for R Shiny App to simulate many data sets and compare aggregate results of statistical designs.

**Data S1.** Data for analysis in Supporting Information S7.

## Data Availability

Data and code are openly available in a public repository that issues datasets with DOIs. See https://doi.org/10.5281/zenodo.13737990.
